# Characterising the Phenotypic Diversity of *Papilio dardanus* Wing Patterns Using an Extensive Museum Collection

**DOI:** 10.1371/journal.pone.0096815

**Published:** 2014-05-16

**Authors:** Martin J. Thompson, Martijn J. T. N. Timmermans

**Affiliations:** 1 Life Sciences Department, Natural History Museum London, London, United Kingdom; 2 Department of Zoology, University of Cambridge, Cambridge, United Kingdom; 3 Division of Biology, Imperial College London, London, United Kingdom; University of Arkansas, United States of America

## Abstract

The history of 20^th^ Century evolutionary biology can be followed through the study of mimetic butterflies. From the initial findings of discontinuous polymorphism through the debates regarding the evolution of mimicry and the step-size of evolutionary change, to the studies on supergene evolution and molecular characterisation of butterfly genomes, mimetic butterflies have been at the heart of evolutionary thought for over 100 years. During this time, few species have received as much attention and in-depth study as *Papilio dardanus*. To assist all aspects of mimicry research, we present a complete data-derived overview of the extent of polymorphism within this species. Using historical samples permanently held by the NHM London, we document the extent of phenotypic variation and characterise the diversity present in each of the subspecies and how it varies across Africa. We also demonstrate an association between “imperfect” mimetic forms and the transitional race formed in the area where Eastern and Western African populations meet around Lake Victoria. We present a novel portal for access to this collection, www.mimeticbutterflies.org, allowing remote access to this unique repository. It is hoped that this online resource can act as a nucleus for the sharing and dissemination of other collections databases and imagery connected with mimetic butterflies.

## Introduction

One of the core questions in evolution biology concerns the origin of morphological novelty – where do new phenotypes come from and what is the nature of the changes which give rise to them? Insect wing patterns are among the most amenable structures for investigating processes determining phenotypic evolution and adaptive colouration [1]–[5] and have provided many textbook examples on the genetics of adaptive change. In particular, wing pattern mimicry in butterflies has been used as a case-study in adaptation [6]–[8].

During the early 20^th^ Century, evolutionary biologists such as Punnet, Fisher, Ford and Dobzhansky were integrating the theories of Mendelian genetics into studies of evolution and biological diversity and using this new perspective to synthesise novel mathematical and theoretical underpinnings of the science [9]–[12]. Throughout the Modern Synthesis, polymorphic mimicry in butterflies was cited repeatedly as offering a corollary of species formation and the origins of evolutionary novelty [13]. In particular, competing interpretations of mimetic butterflies were at the heart of disagreement about the expected step-size of evolutionary change: what is the magnitude of changes which are the basis for novel phenotypes or species [14]–[17]?

**Figure 1:**
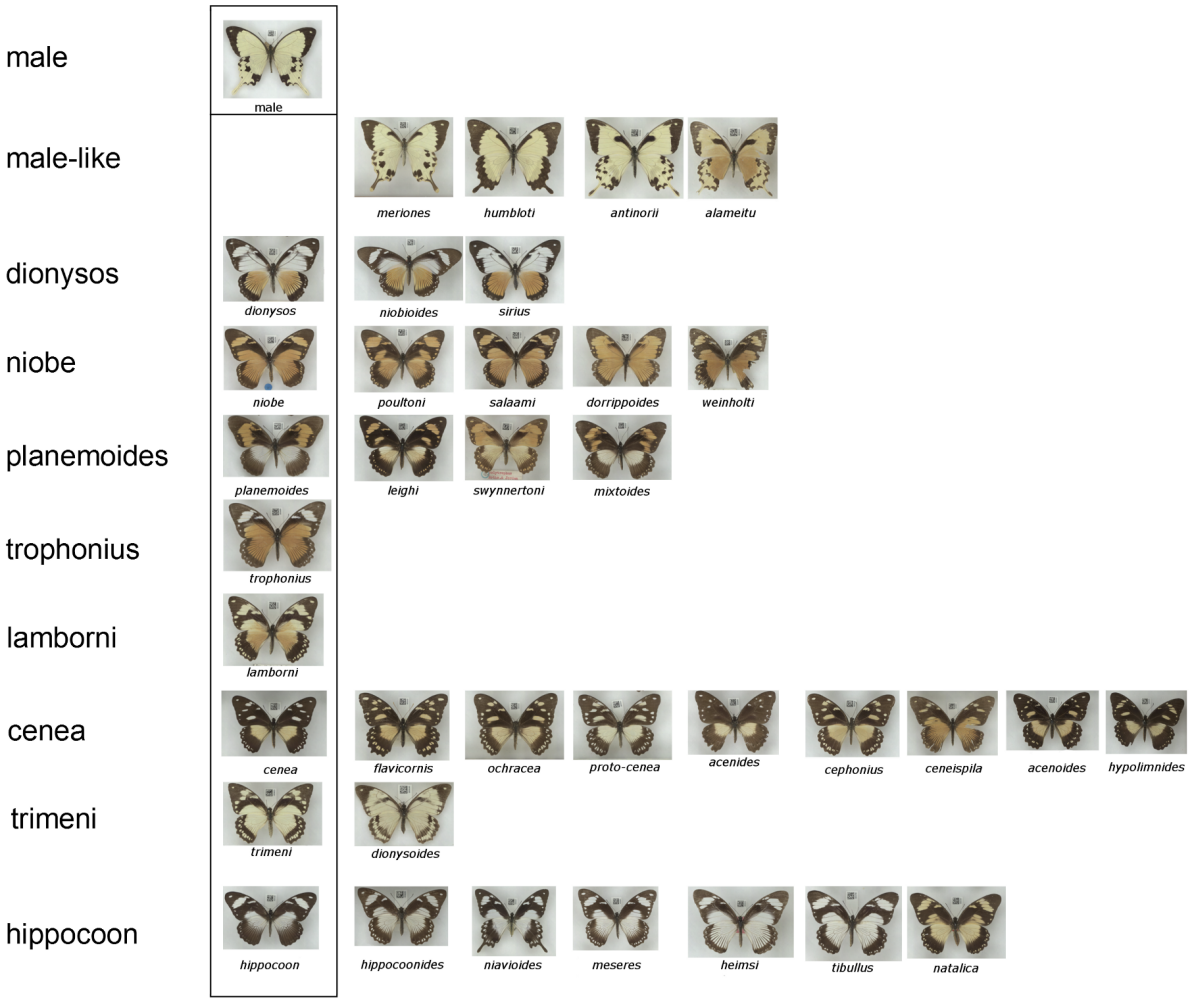
Female morphs of *Papilio dardanus* recognised in this study. Similar looking morphs are grouped into morph groups on each horizontal line, largely following the suggestions of Bernardi et[32]. Forms are present in order of the dominance hierarchy, apart from males and male-like forms (top two rows) and the f. *dionysos* group. In the case of the morph f. *dionysos*, no breeding work exists to support the conclusion of it being an allele at *H*, rather than being due to a second, unlinked locus. Not figured are forms which are not well represented in the NHM London collection, primarily some ‘tailed mimic’ forms of ssp. *P. dardanus antinorii* such as f. *vaccaroi* (tailed variant of f. *cenea*).

At the centre of this debate was a spectacular ‘*exemplar*’ of polymorphic Batesian mimicry [18] the Mocker Swallowtail *Papilio dardanus* Yeats in Brown 1776. Females of this African butterfly mimic members of various Müllerian mimicry rings, largely Danaidae and Acraeini [19], [20]. *P. dardanus* displays a very diverse array of wing patterns (Figure 1) and it is therefore not surprising that many forms were initially described as separate species. It was not until 1869 that it was recognised that these forms did in fact belong to one species [21]–[23].

**Figure 2:**
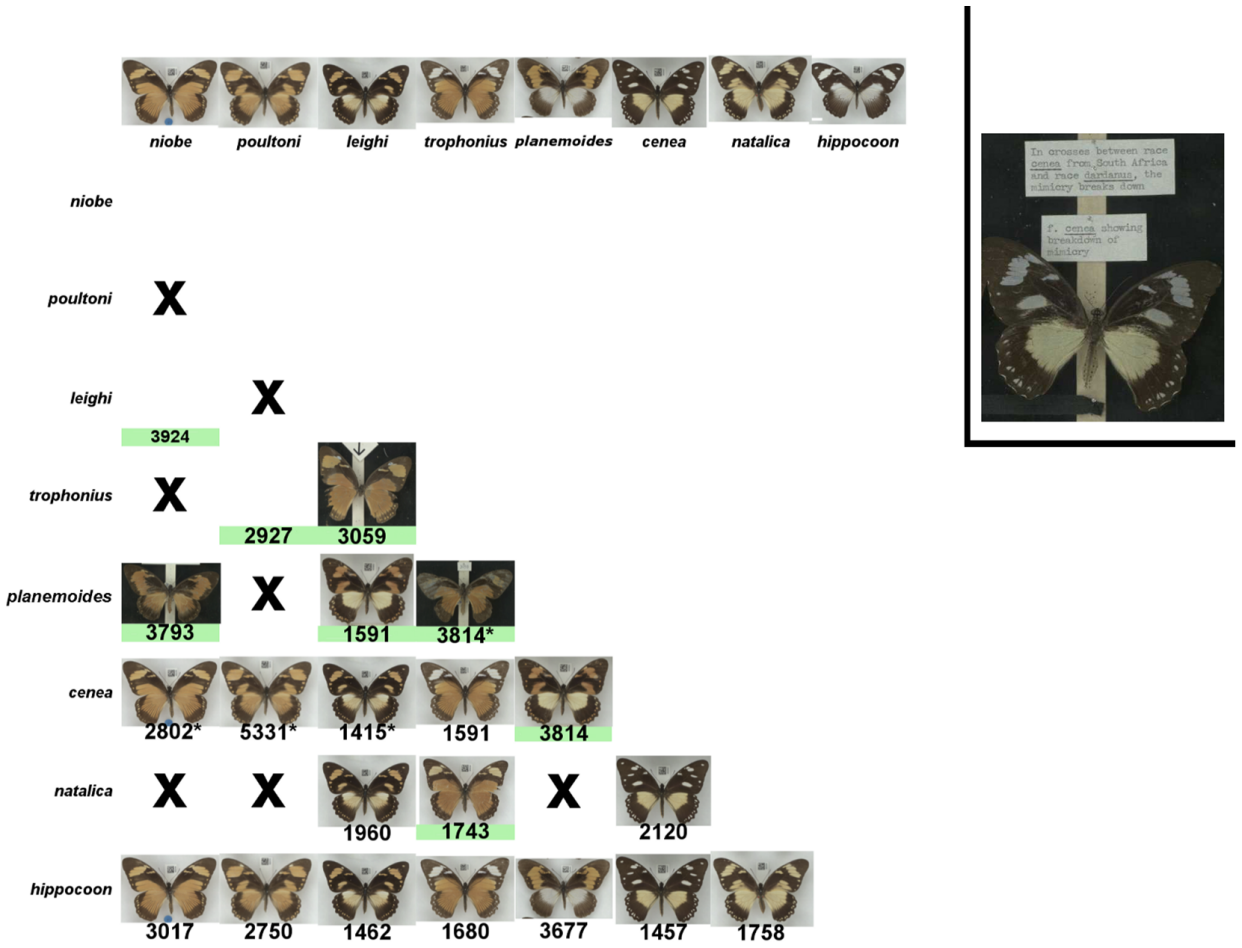
Inheritance of female wing patterns in *Papilio dardanus*. Overview of the crosses performed by Clarke and Sheppard in the 1950s and 1960s. Asterisks indicate crosses with variable outcomes that range from complete dominance to breakdown of mimicry. Green shading indicates incomplete dominance (production of intermediate phenotype). Where possible, intermediates are taken from the Clarke/Sheppard/Turner collection [40], otherwise insects were chosen from wild-caught specimens, which match the descriptions given in the published manuscripts. Most intra-subspecific crosses follow a strict dominance hierarchy, with only a few exceptions. For example, crosses between co-occurring forms f. *trophonius* and f. *natalica* produce butterflies where the dominant phenotype (f. *trophonius*) shows atypical tawny tinted forewing patches. In contrast, inter-subspecific crosses often produced aberrant butterflies. For example, on crossing a *P. dardanus dardanus* f. *cenea* with a male from a West African population of *P. dardanus dardanus*, which lacks this form, the female progeny no longer possessed accurate mimicry of *Amauris echeria*, but displayed an intermediate pattern (inset).

The genetics of wing pattern determination in *P. dardanus* was studied intensively in the 1950s and 1960s by Clarke and Sheppard [24]–[27]. Their investigations built on previous breeding experiments (described in [19], [20]) and the documented inheritance of the various phenotypes were vital for understanding the genetics of mimetic wing patterns in *P. dardanus*. Their controlled crosses, summarised in Figure 2, confirmed that within a single population inheritance of wing pattern generally behaved as a single Mendelian factor with large phenotypic effect and a characteristic dominance hierarchy, consistent with theoretical expectations [28]–[30]. However, when they mixed locally-adapted genetic backgrounds by crossing individuals from different subspecies or from populations with differing morph compositions, the dominance hierarchy between the forms broke down, often with largely unpredictable outcomes (Figure 2). This indicates that in addition to a large single effect locus, wing pattern in *P. dardanus* is affected by unlinked modifier genes that perfect mimetic resemblance to locally co-occurring models [31]. More recent work has identified candidate genes for the mimicry switch locus – the transcription factor *invected* and its paralogue *engrailed* [32].

*P. dardanus* is noted for its variability across Africa [20]. The species is considered to be composed of between 11 and 18 subspecies (Table 1, [33], [34]). Despite extensive interest for over 100 years, we have not yet answered many of the questions initially posed concerning its mimicry. It is unfortunate that much of the existing literature remains relatively inaccessible – in some cases due to its age (1920s–1950s), or its publication in non-English language journals (e.g. [33]), or in defunct or low-circulation journals [19], [35]. Different authors report different numbers of female morphs, which makes obtaining a comprehensive overview of the system difficult (e.g. Grimaldi and Engel [36] mention 5 forms, whereas Clark et al. [32] suggest more than dozen forms exist). Such difficulties are undoubtedly compounded by the fact that some of the names used for *P. dardanus* subspecies are also utilised to describe female forms; this nomenclatural impediment is an unfortunate legacy of earlier works, a fact lamented as early as 1924 [19]. The fact that there are (relatively) few comprehensive image sets of the female forms is also problematic. Such figures as do exist (e.g. Figure 1 in Nijhout, 2003 [37]) typically demonstrate the major differences between the morph groups, but the existence of intermediates and the high levels of diversity within morph groups has not often been emphasised. Of particular note is the possibility for refinement of mimicry to become locally adapted to variations in model pattern [38]. This is well described for the forms f. *hippocoon* and f. *hippocoonides* [39] and it should be recognised that this may exist for other morphs of *P. dardanus*.

**Table 1:** List of subspecies, morph groups and ranges, summarised from Bernardi et al. [32].

Subspecies	Clark & Vogler clade	Range	Morphs
dardanus	Western	Western and central Africa	hippocoon, dionysos, planemoides, trophonius, cenea, niobe, lamborni, niobioides, natalica, mixtoides
tibullus	Eastern	Eastern Africa from Kenya to Mozambique	cenea, trophonius, hippocoonides, salaami, natalica, trimeni, poultoni, lamborni, mixtoides
cenea	Eastern	South Africa	cenea, leighi, trophonius, hippcoonides, salaami, natalica
ochraceana	Western	Mount Marsabit and Mount Nyiru, Kenya	ochracea
flavicornis	Eastern	Mount Kulal, Kenya	flavicornis, atavica
antinorii	Eastern	Ethiopia and South Sudan	antinorii, niavoides, ruspinae, weinholti, vaccaroi
meriones	Indian Ocean	Madagascar	meriones
humbloti	Indian Ocean	Comoros	humbloti
meseres	Eastern	Lake Victoria area, Kenya, Uganda and Tanzania	hippocoonides, cenea, meseres, lamborni, planemoides, salaami, trimeni, mixtoides
polytrophus	Eastern	Central Kenya, East of the Rift Valley	hippocoonides, cenea, meseres, poultoni, lamborni, planemoides, trimeni, swynnertoni, carpenteri, mixtoides
byatti	Eastern	Somalia	
*figinii*		Eritrea	
*alticola*		Ethiopia and South Sudan	
*hodsoni*		Ethiopia and South Sudan	
*sulphureus*		Bioko, Sao Tome and Principe	
*xanthocaudatus*		Rift Valley, Kenya	
*nairobianus*		Kikuyu area, Kenya	

Subspecies in italics are not recognised by all authors, and have not been considered further in the present work. The f. *hippocoon* group is separated into forms related to f. *hippocoon*, f. *hippocoonides* and the intermediate form f. *meseres*. Membership of the Eastern, Western or Indian Ocean clade is given based on Clark & Vogler's analysis of cytochrome b [47].

Much of the contemporary work on this species concerns the molecular characterisation of the mimicry switch locus: how a small genomic region can direct wing pattern development along divergent trajectories leading to widely divergent mimetic patterns, and what we can learn from such a switch locus regarding the evolutionary origin of novel morphology. If we are to understand the evolutionary processes giving rise to morphological diversity, a necessary preliminary step is to fully document and describe the extent of natural variation. As work continues to further characterise and understand the genetic basis and evolutionary biology of wing pattern evolution in *P. dardanus* [32], museum collections will play an important part in documenting the remarkable phenotypic diversity displayed by this species. To this end, we present a thorough analysis of all historical specimens held by the Natural History Museum (NHM) London. All specimens were digitised and databased. Metadata was used for the generation of concise maps detailing the distributions of most of the described subspecies. By making all this information freely available online, we hope to trigger collaborative scientific interest in the “*most interesting butterfly of the world*” [19].

## Materials and Methods

All samples are permanently held by the NHM London under specimen numbers BMNH933783-BMNH937172.

The NHM collections are home to a large number of wild-caught specimens of *P. dardanus*, along with bred series from investigators such as E. G. L. van Someren and C.A. Clarke and P.M. Sheppard [40]. Specimen labels were copied verbatim for all *P. dardanus* and all individuals were given unique identifier labels (voucher numbers) in the form of both a numerical label and a 2D barcode, generated with Qdatamatrix [41]. Individuals were classified into morphs and subsequently organised into groups according to Bernardi et al. [33], with the exception of f. *leighi*, which was included in the f. *planemoides*-group based on its similarity to f. *planemoides* in the crosses of Clarke and Sheppard (summarised in Figure 2).

Drawers of specimens were digitised in the Sackler Biodiversity Imaging Lab at the NHM London using a SmartDrive SatScan machine (Huntingdon, Cambridgeshire, UK) and individuals were cut from the drawer-scan images manually in GIMP 2.8.2 (www.gimp.org). All data were stored in a MySQL database (http://www.mysql.com), accessible via http://www.mimeticbutterflies.org/.

Geocoding was done manually for all specimens, using internet resources (Global Gazetteer 2.2 http://www.fallingrain.com/world/, http://www.mapplanet.com and the Fuzzy Gazetteer http://isodp.hof-university.de/fuzzyg/). Only specimens where an unambiguous match to a location with a linear distance error of less than 40km were used. The geocoded dataset was imported as a point vector file from comma-separated values in ArcMap 10 [42] and projected into an Albers equal-area (Africa) space for validation, plotting and data management. Records were split into separate point vector layers corresponding to assigned morph, morph group or subspecies values. Sampling effort was estimated with a hot-spot analysis of a point density layer constructed in ArcMap 10 [42]. Plotting of Z-values from the Moran's I tool determined the most appropriate distance band for point density and hot-spot (Getis-Ord G_i_*) analyses [43]–[45]. Briefly, varying distance bands were used to calculate Z-values; the distance band with the highest score indicates the scale at which there is the highest clustering of sampled locations. The distance so obtained is therefore an indication of the ‘clumpiness’ of the data and provides an appropriate scale for the hot-spot and point density analyses.

Subspecies were assigned using existing designations where possible, supplemented by extending these designations to nearby areas and using characteristic wing pattern forms in certain subspecies (a combination of male patterning and the use of unique female forms: e.g. f. *tibullus* in subspecies *P. dardanus tibullus* or f. *flavicornis* in subspecies *P. dardanus flavicornis*). Polygons representing inferred distributions of taxa were estimated by constructing a convex hull from the point locality data using the minimum bounding geometry tool in ArcMap 10 [42]. The resulting polygon was clipped using ecosystem and isobioclimate data from USGS data ([46]; United States Geological Survey Africa Ecosystems mapping project, http://rmgsc.cr.usgs.gov/outgoing/ecosystems/AfricaData/). Briefly, ecosystem and isobioclimate data were downsampled to 20 km rectangles using block statistics from USGS raster map and ecosystem or isobioclimate classes were extracted for each specimen point locality locality using the ‘extract values to points’ tool in ArcMap 10 [42]. Ecosystem or bioclimate classes with zero *P. dardarnus* samples were clipped from the convex hull.

## Results

### General summary of the database

All wild caught *P. dardanus* that are preserved in the NHM pinned butterfly collection were digitised and databased. Voucher numbers, sex, subspecies designation and verbatim copied data labels were stored in a MySQL database that can be queried for morph and subspecies via a web-interface available at www.mimeticbutterflies.org. Individual images are also made available on this website. High resolution images are available upon request (e.g. for morphometric analyses).

**Figure 3:**
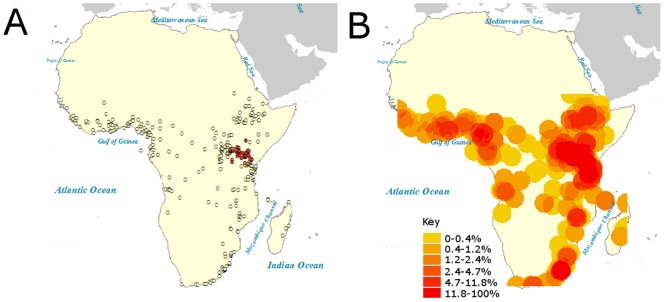
Summary of collection sample density. A) Hotspot analysis showing density outliers. Red dots indicate density more than 2.5 standard deviations above the average. No locations were labelled as outliers with low density. B) Heatmap of sampling density across Africa using circles of radius 280 km. Circles are coloured according to a scale of 7 quantile classes, which are labelled in the key with the proportion of density variation contained within each class.

**Figure 4:**
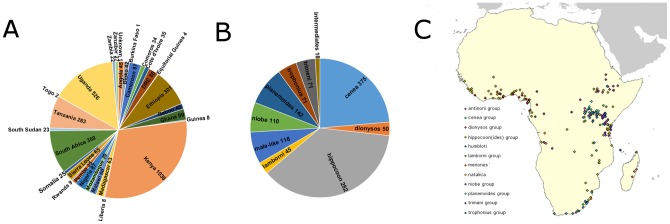
Summary of pinned *Papilio dardanus* preserved by the NHM London. A) Pie chart showing breakdown of NHM London *P. dardanus* collection by country. The islands of Pemba, Zanzibar and Bioko are considered separately from the African mainland. B) Pie chart showing contribution of each morph group among the 1575 female specimens in the collection. Not shown are the 1817 male specimens. The ‘intermediate’ class contains specimens with attributes of more than one morph group, making classification impossible. C) Distribution of point locality data across Africa, labelled by wing pattern.

We assessed the density of collection locations and the levels of coverage using a density and a hot-spot analysis (Figure 3). The results of both these analyses demonstrate that the area of East Africa surrounding Lake Victoria in Uganda and Kenya is much more densely sampled than other areas. This is also borne out in the specimen pie charts (Figure 4A) with Uganda having 528 specimens and Kenya 1035.

The database, which excludes the separate Clark/Sheppard/Turner collection [40] and other bred specimens, currently contains 3395 specimens from 25 countries (Figure 4A–C, note that islands of Pemba, Zanzibar and Bioko have been included separately), with 1576 females (Figure 4). Morphs used in the present study are given along with representative images in Figure 1 (grouped according to Bernardi et al. [33], with the exception of f. *leighi*). The collection contains a considerable number of intermediate, putatively non-mimetic female butterflies (Figure 4) which are believed to result from incomplete dominance or from breakdown of mimetic pattern (similar butterflies were produced in the crosses of Clarke and Sheppard; Figure 2).

### Distributions of forms and subspecies of *Papilio dardanus*

**Figure 5:**
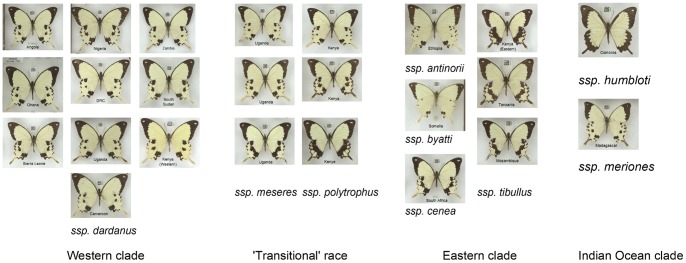
Typical male patterns for each of the subspecies of *P. dardanus*. Variation in male pattern was first used by Jordan [33] to divide *P. dardanus* into sub-species. The variability of pattern in the transitional subspecies *meseres* and subspecies *polytrophus* is noticeable.

*Papilio dardanus* is divided in several subspecies that show a distinct separation in male wing pattern between East and West Africa [34]. This division was later confirmed using genetic analyses [47] and is easily recognisable from the NHM collections material as shown by the males depicted in Figure 5. The museum collection also corroborates that the male patterns of the eastern “races” *P. dardanus tibullus* and *P. dardanus cenea* are very similar.

**Figure 6:**
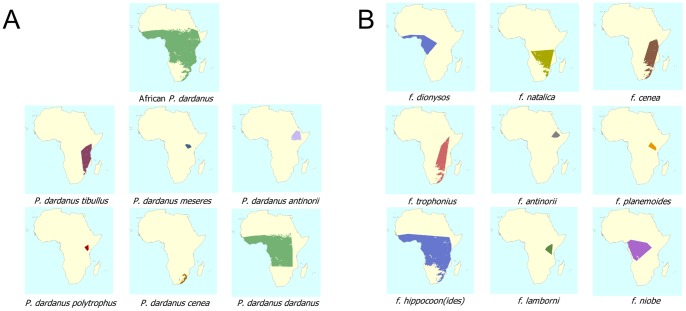
Distribution maps. A) Distribution maps showing the inferred range for the entirety of *P. dardanus* and the various described subspecies. B) Distribution maps showing inferred ranges of the morph groups of female wing patterns in *P. dardanus*. Substantial variation in the extents of subspecies ranges is present, and there is little correspondence between the subspecies ranges (A) and the morph ranges (B).

The dataset reveals that previous subspecies-centred treatments of female morph composition oversimplify the complex phenotypic diversity of *P. dardanus*. Not only are morphs distributed heterogeneously across Africa, but substantial variation exists within each subspecies in terms of the number of morphs co-occurring at sites (Figure 6A and 6B). This is the first time, to our knowledge, that distribution data for the various female forms has been analysed on a continental scale without reference to relatively subjective subspecies boundaries. Most phenotypes are not distributed over the full subspecies ranges, but are found in smaller areas only (Figure 6B): for example, f. *dionysos* is restricted to Western Africa and Cameroon and shows little overlap with other forms except for f. *hippocoon*, whilst the subspecies *P. dardanus dardanus* has its eastern limit in western Kenya and is highly polymorphic in this area.

In some cases, however, subspecies can be characterised by the presence of unique morphs. For example, f. *antinorii* (a male-like female phenotype) is endemic to subspecies *P. dardanus antinorii* from Ethiopia. This subspecies also contains unique tailed variants of the mimetic forms f. *trophonius* (f. *ruspinae*), f. *poultoni* (f. *weinholti*), f. *hippocoonides* (f. *niavioides*) and f. *cenea* (f. *vaccaroi*). The scarcity of many of these tailed forms prevents a systematic analysis of their distribution, but our data suggests that all are found within the range of *P. dardanus antinorii*. Unfortunately, it was not possible to georeference any specimens of *P. dardanus byatti*. This subspecies is likely of interest with respect to the evolution of polymorphic mimicry in female *P. dardanus*, it being a mainland subspecies with only male-like females known (Vane-Wright, pers comm).

### Imperfect mimics and the ‘transitional race’

**Figure 7:**
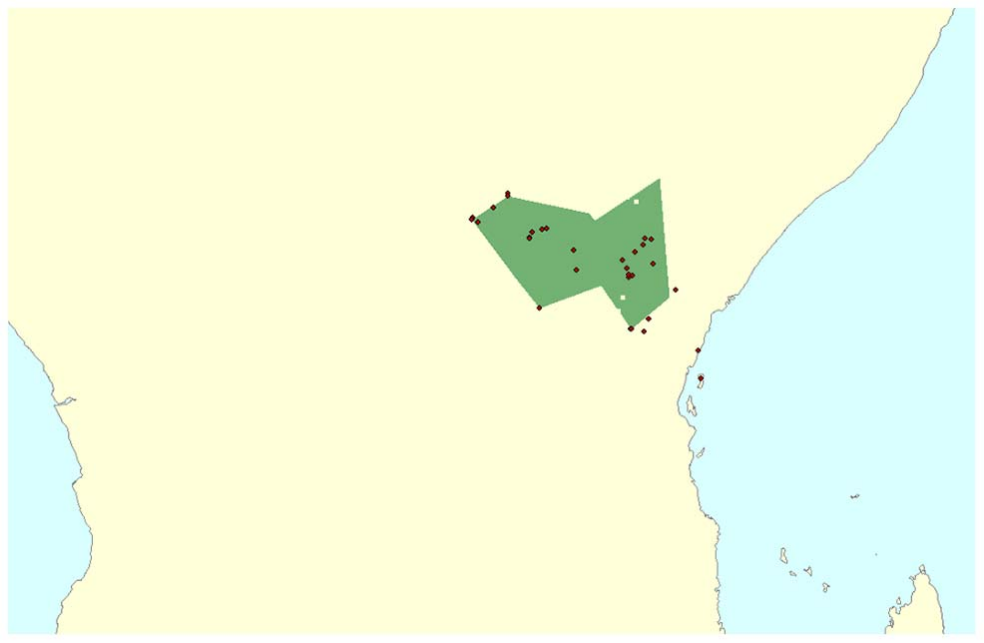
Localities of intermediate forms. Point locality for intermediate forms and imperfect mimics overlain over inferred polygon for subspecies *polytrophus* and *meseres*. Female forms plotted are: f. *trimeni*, *f. dorrippoides*, f. *dionysoides*, f. *proto-*cenea, f. *mixtoides*, f. *swynnertoni*, f. *carpenter* and f. *lamborni*, and intermediates between f. *lamborni* and other forms.

We used the NHM collection to investigate whether imperfect forms are over-represented in specific subspecies. We specifically focussed on the populations of around Lake Victoria and Mount Kenya as they are often described to contain a high proportion of ‘imperfect’ mimics that show a mix of patterns from several forms, or contain traces of the fluorescent yellow male pigment [19], [20], [25], [48]. These subspecies, intermediate between Eastern and Western African lineages [34], [49], [50], have been suggested to be of hybrid nature occurring as they do at a contact zone between the West African subspecies [37]. We find that ‘imperfect’ forms are indeed associated with these two subspecies. Figure 7 shows a point-in-polygon overlay of point locality data of ‘imperfect’ individuals (the intermediate individuals in Figure 1 along with morph groups f. *lamborni* and f. *trimeni* and the individual imperfectly mimetic forms f. *dorrippoides*, f. *mixtoides* and f. *proto-cenea*) alongside the inferred polygon for the extent of the two ‘transitional’ subspecies *P. dardanus meseres* and *P. dardanus polytrophus*. Out of 145 imperfect mimetic specimens which could be geocoded, 131 were collected from within the extent of subspecies *P. dardanus meseres* and *P. dardanus polytrophus*. The remaining 14 were all from locations in Kenya (particularly towards the Kenyan coast) and Eastern Uganda (Figure 7).

## Discussion

Studies on mimetic butterflies have the potential to shed light on questions going to the core of contemporary evolution biology. These insects have been studied for over 100 years, informing our knowledge of the origin of novel traits and species, and the genetic architecture underlying adaptation. In particular, mimetic butterflies exemplify the multi-dimensional nature of adaptation to different environments. Polymorphic mimicry requires accurate matching of diverse wing patterns differing in several characters, likely determined by several genetic loci. However, the processes of recombination and the independent assortment of chromosomes mean that the loci involved may be broken up, leading to inaccurate mimicry, unless they are closely linked. The answer to this paradox is found in the concept of supergenes [51], tightly linked blocks of multiple co-adapted genetic elements. *Papilio dardanus* is renowned for the morphological diversity of its mimetic females and has been central to the development of the supergene theory. However, although supergenes have recently been shown to underlie polymorphism in other mimetic butterflies [6], [8], their existence in *P. dardanus* remains unconfirmed. To promote *P. dardanus* as a study system, we present a systematic overview of variation displayed within the species accompanied by distribution maps. We expect this work to facilitate future research on the evolutionary genetics underlying mimicry by providing a concise overview of the variation displayed and by allowing identification of areas of interest within the range of *P. dardanus*. The presented distribution maps show that the degree of polymorphism present at a site varies greatly and the choice of which population to study will undoubtedly affect the ease with which we may come to understand the genetic control of polymorphic mimicry in this species.

Although the mimetic females of *P. dardanus* have received most scientific attention, subspecies have largely been defined on the basis of differences in male wing pattern. In 1905, Jordan [33] divided *P. dardanus* from the African continent (whilst excluding the populations in North East Africa) into five ‘races’, and described a clear separation between the western subspecies (*P. dardanus dardanus*) and the three eastern ones (*P. d. cenea*, *P. d. tibullus* and *P. d. polytrophus*). His fifth subspecies occupied the contact zone and showed intermediate traits (*P. d. meseres*, [50]). Jordan reports that the separation between the three eastern subspecies is less clear-cut and shows much morphological overlap – the male wing patterns of *P. d. tibullus* and *P. d. polytrophus* and the male genitalia of all three eastern subspecies are similar [49]). The collection material presented here is consistent with these subspecies descriptions. It shows the clear divergence between the East and West African populations (Figure 5) and corroborates that the eastern “races” share male wing pattern phenotypes (see also [33]). We do not believe that the existing subspecies designations should be discarded however. The subspecies as described reflect populations evolving somewhat independently of others, as reflected by the documented differentiation in male wing patterns and genitalia. The subspecies boundaries in *P. dardanus* are porous, allowing morph distributions to evolve independently in most cases, and precluding the accumulation of detectable genetic differentiation.

Female wing pattern variation in *P. dardanus* is often presented as following a clear-cut Mendelian hierarchy, a prerequisite of accurate polymorphic mimicry: intermediate phenotypes are non-mimetic and lack protection from predation [52]. The collection reveals this to be a simplification, and in fact the phenotypes arising from a mating depend on a multitude of factors including the particular alleles and the genetic background at loci other than the single, large effect mimicry switch (Figure 2). Even in single populations, intermediate heterozygote phenotypes exist, for example. f. *salaami* in *P. dardanus cenea* [24]. Such additional complexity is, we feel, often overlooked and yet it may be of great future interest as it allows insight into the role of modifier genes in the perfection of polymorphic mimicry and is an example of the evolution of dominance relationships.

Under the prevailing theory of secondary ‘modifier’ loci alternately perfecting the mimicry in different subspecies, contact zones where genetic backgrounds become admixed should display mimicry and dominance breakdown. This hypothesis is supported by our results, which show that the *P. dardanus meseres* and *P. dardanus polytrophus* populations, which are found in-between the diverged eastern and western mainland lineages [47], have very variable wing patterns (Figure 2, Figure 7). Almost all deviating phenotypes were collected from this region and the boundaries between subspecies are here indistinct, overlapping with *P. dardanus dardanus* (for *P. dardanus meseres*) or *P. dardanus tibullus* (for *P. dardanus polytrophus*).

Analyses of these ‘transitional’ populations may therefore shed light on the role of unlinked modifier loci in determining accurate mimetic wing patterns, similar to the insight gained from studying hybrid zones between *Heliconius* races [53]. Ongoing work to sequence and characterise genomes from several subspecies of *P. dardanus* is already underway. From the results presented here, we predict that the subspecies of *P. dardanus meseres* and *P. dardanus polytrophus* likely facilitate gene flow between eastern and western lineages. Fixed divergence between these lineages will be low, apart from at loci under disruptive selection (such as mimicry modifier loci) which would show a strong signal of differentiation [54], [55]. Such a situation would be strongly reminiscent of the situation of the multiple, parapatric races of *Heliconius* species, which are differentiated from one another despite genome-wide gene flow [56].

An alternative hypothesis to explain the ‘breakdown’ of mimicry in this ‘transitional’ region is a reduction in effective selection for mimicry due to either a reduction in predation pressure or to local scarcity of distasteful model species [20], [57]. The validity of the two hypotheses could be tested with the help of the distribution data, which will allow the identification of further contact zones between the two mainland lineages (e.g. around Lake Malawi, as suggested by Turner [49] on the basis of the genitalia of a single male butterfly) that may, or may not show breakdown in mimicry.

This study highlights the continuing importance of museum collections and demonstrates the utility of digitising collection information. The digitisation of the NHM London *P. dardanus* collection allows us to obtain detailed information on the biology and ecology of this species. However, we must recognise the incomplete nature of any collection: for example, as shown in Figure 3 large gaps exist in the distribution of sampled locations and demonstrates the variation in sampling density across Africa. By making the NHM London collection information openly available online, it may eventually be possible to fill in some of the spatial and/or temporal sampling gaps through the incorporation of specimens held by a number of different institutions. Yet, we believe it is already possible to begin to synthesise a more complete picture of the biology of *P. dardanus*. For example, precise information on the distribution of each of the mimetic phenotypes could be correlated with the distribution of their models to test the mimicry system at the ecological level. If shaped by natural selection, correlations between the ranges of mimetic forms and their models are expected to be strong. Discrepancies might indicate recent range shifts or other ecological factors being of importance (e.g. the absence of predators). The observed statistical patterns will assist molecular studies and potentially explain genetic changes, such as these indicating recent selective sweeps or population bottlenecks.

The staggering diversity of *Papilio dardanus* continues to captivate evolutionary biologists. The systematic overview presented here unlocks a single museum collection and reveals an unequal distribution of morph classes over sub-Saharan Africa. It also exposes the generally overlooked abundance of imperfect phenotypes. Poulton once noted on the origin of phenotypes in *P. dardanus* that “*the study is hindered at the outset by a complicated nomenclature*” [19] Here we followed the classification of Bernardi et al. [33], and grouped the female forms in 9 classes. This, however, remains an artificial classification, merely based on resemblance rather than the fundamental genetics of the system, ignoring the possibility that some phenotypes may have evolved convergently. Future molecular work on this intraspecific wing pattern radiation will reveal whether similar-looking colour patterns are indeed genetically related and share similar evolutionary histories or have arisen multiple times under strong mimicry selection.
