# Correction: Characterising the phenotypic diversity of *Papilio dardanus* wing patterns using an extensive museum collection

**DOI:** 10.1371/journal.pone.0105826

**Published:** 2014-08-13

**Authors:** The PLOS ONE Staff

The corresponding author indication is incorrect. The correct corresponding author is Martijn Timmermans (mjtntimmermans@gmail.com). The publisher apologizes for this error.
